# Strain-Dependent Cheese Spoilage Potential of *Clostridium tyrobutyricum*

**DOI:** 10.3390/microorganisms8111836

**Published:** 2020-11-22

**Authors:** Lucija Podrzaj, Johanna Burtscher, Franziska Küller, Konrad J. Domig

**Affiliations:** 1 Institute of Food Science, University of Natural Resources and Life Sciences Vienna (BOKU), 1190 Vienna, Austria; lucija.podrzaj@boku.ac.at (L.P.); franziska@kueller.eu (F.K.); konrad.domig@boku.ac.at (K.J.D.); 2 Austrian Competence Centre for Feed and Food Quality, Safety and Innovation (FFoQSI GmbH), Technopark 1C, 3430 Tulln, Austria

**Keywords:** *Clostridium tyrobutyricum*, spore, spoilage, late blowing, cheese

## Abstract

*Clostridium tyrobutyricum*, a Gram-positive, anaerobic, spore-forming bacterium, is considered as one of the main causative agents for spoilage of hard and semihard cheeses. Growth of *C. tyrobutyricum* in cheese is critically influenced by ripening temperature and time, pH, salt and lactic acid concentration, moisture and fat content, and the presence of other microorganisms. Previous studies revealed high intraspecies diversity of *C. tyrobutyricum* strains and variable tolerance toward pH, temperatures, and salt concentrations. These findings indicate that strain-dependent characteristics may be relevant to assess the risk for cheese spoilage if clostridial contamination occurs. In this study, we aimed to compare the phenotypes of 12 *C. tyrobutyricum* strains which were selected from 157 strains on the basis of genotypic and proteotypic variability. The phenotypic analysis comprised the assessment of gas production and organic acid concentrations in an experimental cheese broth incubated at different temperatures (37, 20, and 14 °C). For all tested strains, delayed gas production at lower incubation temperatures and a strong correlation between gas production and the change in organic acid concentrations were observed. However, considering the time until gas production was visible at different incubation temperatures, a high degree of heterogeneity was found among the tested strains. In addition, variation among replicates of the same strain and differences due to different inoculum levels became evident. This study shows, that, among other factors, strain-specific germination and growth characteristics should be considered to evaluate the risk of cheese spoilage by *C. tyrobutyricum*.

## 1. Introduction

The contamination of cheese milk with endospores of butyric acid-producing clostridia represents a major concern for cheese producers. Although milk does not provide suitable growth conditions, clostridial endospores may encounter favorable conditions for germination and outgrowth during ripening of hard and semihard cheese. This metabolic activity leads to pronounced cheese spoilage [1]. The production of excessive amounts of gas and butyric acid during growth results in blown cheeses of inferior sensory quality and financial losses for producers [2]. Several clostridial species, mainly *Clostridium tyrobutyricum, Clostridium butyricum*, *Clostridium sporogenes*, and *Clostridium beijerinckii*, have been isolated from blown cheese. The species *C. tyrobutyricum* is considered to be the primary cause of severe late blowing defects (LBD) [3,4,5,6]. Among strains of this species, however, significant differences have been observed. On the one hand, genetic heterogeneity has been illustrated using strain typing methods; on the other hand, some studies characterized phenotypic variation among *C. tyrobutyricum* strains [7,8,9]. For instance, Silvetti et al. demonstrated that pH, salt concentrations, and temperature can affect spore germination, growth, gas production, and reduction potential of vegetative cells and spores of clostridia in an enriched milk medium [10]. They observed that a temperature below 15 °C prevented the gas production by vegetative cells, while a combination of temperature, pH, and salt concentration was needed to prevent germination of spores and subsequent gas production. Moreover, Ruusunen et al. observed varying tolerance to changes of pH, temperature, and salt concentrations among 10 *C. tyrobutyricum* strains in tryptone–peptone–glucose–yeast extract and reinforced clostridial broth [11]. Garde et al. concluded that the production of gas and butyric acid in milk and Bryant and Burkey medium (BB) seems to be strain-dependent [12]. The results obtained in the course of these previous studies raised further questions about the cheese spoilage potential of *C. tyrobutyricum*. Does the genetic diversity among the strains reflect phenotypic diversity and vice versa? If the spoilage potential is strain-dependent, can the same behavior of a strain be expected in repeated experiments? To the best of our knowledge, these issues have not been specifically addressed in previous studies. Hence, the aim of this study was to further elucidate the strain-dependent cheese spoilage potential of *C. tyrobutyricum*. For this purpose, we assessed the genetic diversity of 157 clostridial strains. A subset of 11 genetically distinct strains and the type strain were selected for phenotypic analyses by assessing gas and organic acid production at different incubation temperatures in an experimental cheese broth.

## 2. Materials and Methods

### 2.1. Assessment of the Genetic and Proteotypic Diversity of the Test Strain Set

A total of 157 *C. tyrobutyricum* strains of the culture collection of the Institute of Food Science of the University of Natural Resources and Life Sciences Vienna were used in this study. The strain set included four strains from the German Collection of Microorganisms and Cell Cultures, i.e., DSM 2637 (type strain; named Cl_20 in this study), DSM 663 (Cl_14), DSM 664 (Cl_15), and DSM 1460 (Cl_2), three strains isolated from NIZO strain BZ 15 (Cl_25, Cl_29, and Cl_51), three strains from Agroscope in Switzerland, i.e., FAM1559 (Cl_33), FAM25158 (Cl_52), and FAM25159 (Cl_53), 83 strains that were isolated from 27 spoiled cheese samples from eight production locations (named A–H) [6], and 64 isolates from 64 raw milk samples collected from three production locations (named A–C) [13]. First, the diversity among the 157 strains of *C. tyrobutyricum* was assessed using MALDI-TOF MS (matrix-assisted laser desorption ionization-time of flight mass spectrometry) and hexaplex-PCR typing according to the procedures described by Burtscher et al. [8]. Data preprocessing and UPGMA (unweighted paired-group method with arithmetic mean) clustering were performed using the software BioNumerics v7.6.3. (Applied Maths, Ghent, Belgium).

According to the genotypic and proteotypic results, 12 representative strains were selected for further phenotypic analyses. A description of the strain selection process is provided in Section 3.1. An overview of the 12 selected *C. tyrobutyricum* strains is presented in Table 1.

### 2.2. Spore Suspension Production and Cultivation Conditions

*C. tyrobutyricum* strains were isolated from cryo-cultures and inoculated into 5 mL of reinforced clostridial medium (RCM: 10 g of meat extract, 10 g of tryptone, 3 g of yeast extract, 10 g of d-(+)-glucose anhydrous, 1 g of starch, 5 g of sodium chloride, 3 g of sodium acetate, and 0.5 g of l-cysteine hydrochloride monohydrate per liter) and incubated for 72 h at 37 °C in anaerobic jars (using a gas mixture containing 80% N_2_, 10% CO_2_, and 10% H_2_; Don Whitley Scientific, West Yorkshire, UK). Then, spore suspensions were produced in triplicate as follows: 1 mL of the grown *C. tyrobutyricum* culture was inoculated into a tube containing 40 mL of peptone yeast glucose broth (PYG: 20 g of tryptone, 10 g of yeast extract, 10 g of d-(+)-glucose anhydrous, 0.4 g of sodium bicarbonate, 0.04 g of monopotassium phosphate, 0.04 g of dipotassium phosphate, 0.008 g of l-cysteine hydrochloride monohydrate, 0.008 g of magnesium sulfate heptahydrate, 0.01 g of hemin, and 0.01 g of vitamin K1 per liter) and incubated for 10 days at 30 °C under anaerobic conditions using the gas mixture described above. After pasteurization at 80 °C for 20 min in a water bath, the tubes were incubated for 3 more days at 30 °C under anaerobic conditions. After a centrifugation step (1300× *g*, 15 min, 4 °C), spore pellets were washed by resuspending them in 10 mL of sterile deionized water and centrifuged again at the same conditions. The washing procedure and centrifugation were repeated twice. The resulting spore pellets were resuspended in 10 mL of sterile deionized water. Spore production and growth was checked under the microscope after spore staining using the dyes crystal violet and safranin. Afterward, the three spore suspensions of a strain were combined into one tube and pasteurized at 80 °C for 20 min.

To determine the spore count, spore suspensions and decimal dilutions thereof were plated in duplicate on reinforced clostridial agar (RCA, Merck, Germany) and incubated at 37 °C under anaerobic conditions. After 7 days, colonies were counted and expressed as colony-forming units (CFU) per mL (Table 1). To confirm the assignment to the species *C. tyrobutyricum*, three grown colonies were randomly selected and identified using MALDI-TOF mass spectrometry (MS), using a Microflex LT instrument and MALDI Biotyper software (Bruker Daltonik, Bremen, Germany) according to the manufacturer’s instructions. The presence of spores was visualized and captured by phase-contrast microscopy using an Olympus BX41 (Olympus, Tokyo, Japan) microscope with a phase-contrast objective (Figure S1, Supplementary Materials). Magnification was accomplished with a 100× oil immersion objective and 10× ocular magnification, resulting in a total magnification of 1000×.

### 2.3. Growth and Gas Production at Different Temperatures

For the assessment of growth and gas production, an enriched milk medium (EM) described by Silvetti et al. was adapted to create growth conditions that are similar to the circumstances present during ripening of typical Austrian hard cheese and other hard cheese types that are usually affected by clostridia [10,14,15,16,17]. In brief, we aimed for a medium containing sodium lactate and acetate as carbon sources, 1% sodium chloride, with a pH of 5.4. For this purpose, reconstituted skim milk (100 g per liter) was supplemented with 10 g of yeast extract, 56 g of sodium lactate (60% *w*/*w*), 10 g of sodium acetate, 10 g of sodium chloride, and 2 g of l-cysteine per liter. The pH value of the medium was adjusted to 5.4 using sterile filtered 1 N HCl.

Before inoculation, if necessary, spore suspensions were diluted to reach final concentrations from 10^2^ to 10^3^ spores per mL of EM. Three strains were prepared in three different concentrations (10^2^, 10^3^, and 10^4^ spores per mL of EM), in order to specifically examine the influence of spore concentration. Next, nine aliquots of 1 mL of each spore suspension were centrifuged (1300× *g*, for 15 min, at 4 °C) and pellets were resuspended in 1 mL of prepared EM. Resuspended pellets were added to glass tubes containing 4 mL EM. To generate anaerobic conditions, the tubes were sealed with a plug consisting of 2.5 mL of melted Vaseline–paraffin. Blank controls were prepared with no spores added, but were otherwise subjected to the same process as the experimental tubes. In the next step, the tubes were subjected to a heat treatment, which resembles the procedure used in Austrian hard cheese production [15]. During the first step, the tubes were heated at 32 °C for 90 min in a water bath, corresponding to conditions under which rennet coagulation occurs. Then, the water bath was heated to 52 °C and the temperature was held for additional 20 min at 52 °C, mimicking the scalding step. Lastly, the tubes were cooled down to room temperature.

Gas production and changes in the EM by clostridial spores were evaluated at three different temperatures: 37, 20, and 14 °C. The temperature of 37 °C represents the optimal growth temperature of clostridia [18]. The second temperature of 20 °C was chosen according to the production process of Swiss Emmental cheese, in which the cheese is first ripened at a temperature of ~20–24 °C for a certain period of time, and then stored at 10–13 °C [19]. Lastly, the temperature of 14 °C was chosen according to ripening temperatures of Austrian mountain cheese such as Vorarlberger Bergkäse and Tiroler Bergkäse [15,16]. Clostridial spores of each strain were incubated at each temperature in triplicate.

During incubation, the tubes were inspected daily for gas production by observing the plug displacement. When, within a set incubated at the same temperature, at least six out of 12 strains showed gas production in at least one replicate, the incubation was interrupted. At 14 °C, however, only four out of 12 strains showed gas production after an incubation of 120 days. Considering the minimum ripening time of 3 months for Austrian mountain cheese, the samples were analyzed at this time point [15]. The tubes were photographed, and the lifted plug height was measured with a ruler. Consequently, 2 mL of the test liquid was harvested from each tube and destined to chemical analysis. The incubation of tubes without gas production was continued until gas production occurred or until the end of the incubation period (37 °C: 150 days; 20 °C and 14 °C: 170 days).

### 2.4. Chemical Analysis

The pH values of harvested test liquids were determined in duplicate using a pH meter Lab 845 (SI Analytics, Mainz, Germany) equipped with an N 6000 BNC electrode (SI Analytics, Mainz, Germany).

Concentrations of organic acids (lactic, acetic, propionic, and butyric acid) in harvested test liquids were determined by high-performance liquid chromatography (HPLC). Cell-free supernatants were prepared from harvested test liquids by centrifugation at 6000× *g* for 15 min at 4 °C. To achieve better precipitation, 150 µL of Carrez solution I (15 g of potassium hexacyanoferrate(II) trihydrate dissolved in 100 mL of distilled water) and 150 µL Carrez solution II (30 g of zinc sulfate heptahydrate diluted to 100 mL distilled water) were added to the test liquid, and then the tubes were centrifuged at 15,000× *g* for 30 min at 4 °C. The supernatant was filtered through a 0.2 mm nylon membrane filter (Carl Roth, Karlsruhe, Germany) and stored at −20 °C until use. Before the HPLC measurement, prepared supernatants were thawed at room temperature, heated for 5 min at 99 °C, and centrifuged at 16,000× *g* for 10 min. Supernatants were transferred into a fresh tube. The samples were injected (volume 20 µL) and eluted with 2.5 mM sulfuric acid at 60 °C and a flow rate of 0.5 mL·min^−1^ on a 300× 7.8 mm ion exchange column HPX-87H Aminex (Bio-Rad, Veenedaal, The Netherlands) in a HPLC system (Dionex Ultimate 3000, Thermo Fisher, Dreieich, Germany), consisting of a WPS-3000 Well-plate autosampler, an LPG-3600 gradient pump, an RS column oven, and a Rapid Separation Diode Array detector at 210 nm for lactic, acetic, propionic, and butyric acid. The HPLC was controlled using the Chromeleon^®^ Software (Version 7.2, Thermo Fisher Scientific, Dreieich, Germany). For obtaining the calibration curves, a mixture of the standards of selected concentrations was injected into HPLC. After injection of the samples, chromatographic peaks were identified by comparing the retention times of the sample with those of the known standards. Results were expressed as net production with respect to the organic acid content in EM (blank control).

### 2.5. Statistics

Statistical analysis of data was performed by means of SPSS software (version 24, SPSS Inc. Chicago, IL, USA). Shapiro–Wilk’s test was used to verify normality and Levene’s test was used to verify homogeneity of variances. Spearman’s correlation coefficients were obtained to investigate potential linear correlations among pH, organic acids, and gas production. Student’s *t*-test was used to detect significant differences among isolates. Comparison of means of pH was carried out by Tukey’s test, with significance assigned at *p* < 0.05.

## 3. Results and Discussion

### 3.1. Genotypic and Proteotypic Diversity

**Figure 1:**
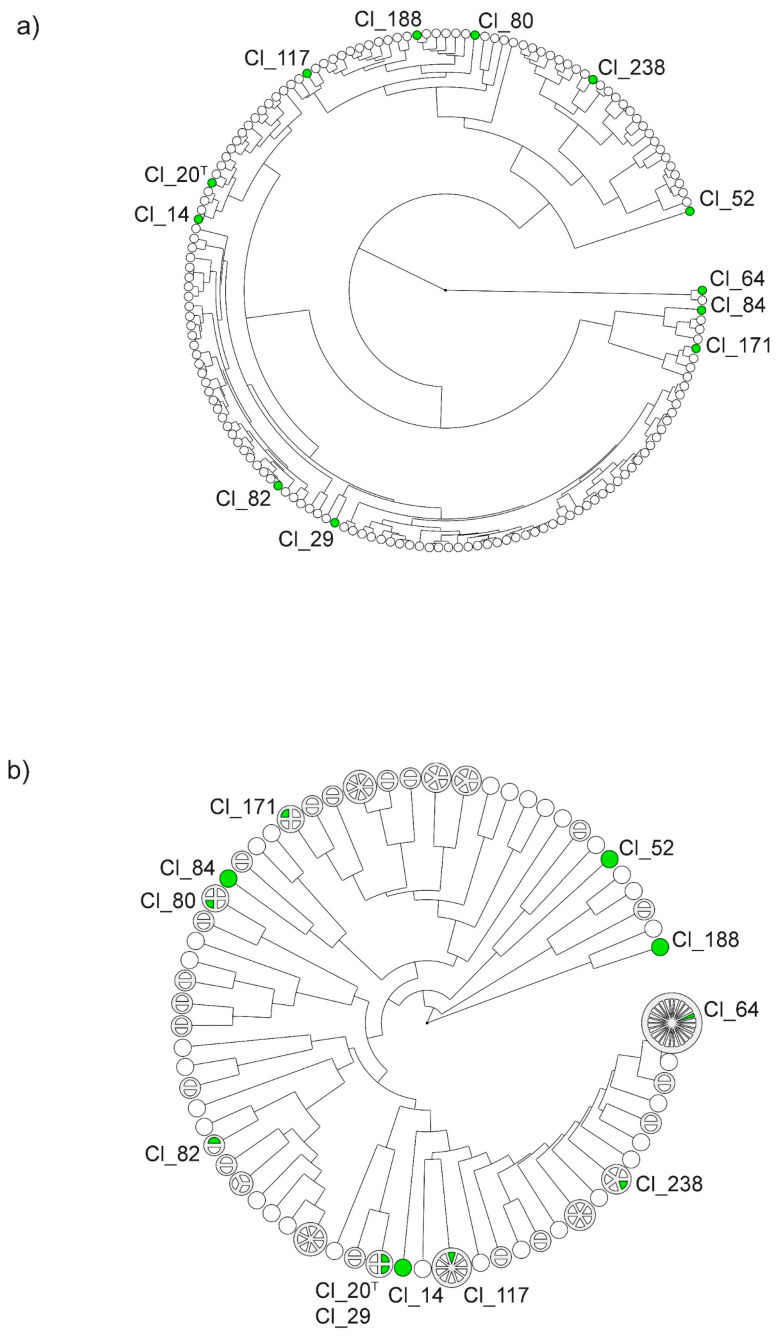
Dendrograms based on matrix-assisted laser desorption ionization-time of flight mass spectrometry (MALDI-TOF MS) spectra (**a**) and hexaplex-PCR band patterns (**b**) in a circular layout. The dendrogram was constructed using the unweighted paired-group method with arithmetic mean (UPGMA). The branch length indicates similarity between different strains and clusters. Each disc symbolizes a strain. Strains that yielded 100% similarity in the cluster analysis were aggregated into larger discs containing segments for each strain (**b**). Strains that were selected for phenotypic analyses are marked in green. ^T^ Type strain.

The obtained dendrograms in Figure 1 illustrate the diversity of the 157 clostridial strains in a circular layout on the basis of the spectra obtained using MALDI-TOF MS (a) and band patterns obtained using hexaplex-PCR (b). Twelve representative strains were selected for further phenotypic analyses. For the selection, the samples in each dendrogram were grouped into clusters. At similarity levels of 80%, the strains grouped into 11 clusters according to their hexaplex-PCR band patterns and into five clusters according to their protein spectra. The lower diversity observed among the MALDI-TOF MS spectra compared to hexaplex-PCR band patterns is consistent with results obtained in a previous study by Burtscher et al. [8]. Generally, the dendrograms obtained using the two typing methods yielded different arrangements of strains. However, some congruence was observed between the two methods. Strains Cl_52 and Cl_84, for instance, clustered separately from other strains in both typing analyses and, therefore, were selected for further assessment of gas and organic acid production. In contrast, the type strain Cl_20 and strain Cl_29 were indistinguishable using hexaplex-PCR and also showed similar protein spectra. Hence, Cl_20 and Cl_29 were selected as representative samples of similar strains. Subsequently, the dendrograms were screened for strains which were evenly distributed across the clusters in both dendrograms. Afterward, the metadata of the remaining strains were checked, and the sample set was finally narrowed down to a subset of 12 strains, which included the type strain (Cl_20 or DSM 2637) and strains, which were isolated at various sampling times from different food sources (cheese types and raw milk) and different production locations.

### 3.2. Gas Production

Gas is one of the major products of butyric acid fermentation, causing swelling, cracks, slits, and irregular eye formation in hard and semihard cheeses [1]. Table 2 provides the number of days until gas production by *C. tyrobutyricum* strains was observed in EM at incubation at 37, 20, and 14 °C. Ten and 11 out of 12 strains were able to produce gas at 37 and 20 °C, respectively. For all strains (except for Cl_64 at 37 °C) at 37 and 20 °C, the results coincided insofar as all three replicates of one strain were either positive or negative. At 14 °C, four out of 12 strains produced gas. In contrast to the higher temperatures, only one strain, namely, Cl_84, showed gas production in all replicates. The average time span between inoculation and observed gas production was 5.5 ± 2.4 days at 37 °C, 30.8 ± 10.3 days at 20 °C, and 108.2 ± 21.4 days at 14 °C. Although the temperature of 14 °C inhibited gas production of most of the strains, it did not entirely prevent gas production. It is evident, however, that lower incubation temperatures delayed the gas production of *C. tyrobutyricum* in EM. Previous in vitro studies also reported the influence of low ripening temperatures on *C. tyrobutyricum* [10,11]. However, Silvetti et al. reported a lower number of days until gas production was obvious under similar conditions [10]. This can be explained by the higher number of spores that were inoculated (10^5^ to 10^7^). Ruusunen et al. observed that a temperature lower than 10 °C inhibited the growth and gas production of some *C. tyrobutyricum* strains [11]. The influence of lowering storage temperatures on the gas formation of other clostridia, such as *C. sporogenes* and *C. beijerinckii*, in culture media and cheese has also been reported [10,20]. In a recent study, Morandi et al. reported a decrease of the presence of *C. tyrobutyricum* spores and consequent late blowing defects in Valtellina Casera PDO (protected designation of origin) cheese during aging at 8 °C, while growth was observed at 13 °C [21].

Interestingly, all replicates of 11 out of 12 strains were able to produce gas at 20 °C, suggesting that the tested *C. tyrobutyricum* strains may have adapted to lower temperatures in the dairy environment (i.e., dairy farm environment, conditions during cheese ripening and storage).

The ability of *C. tyrobutyricum* spores to produce gas even at low ripening temperatures emphasizes the importance of precise control of ripening conditions during cheese manufacture. This finding may be important in the production of traditional raw milk cheese. Of particular importance are some PDO cheese types, for which silage feeding is often prohibited and a traditional production process is required. Moreover, for these cheeses, physical treatments and additives used against clostridial spores are often prohibited [22]. For other cheeses, however, clostridial spores may be reduced via centrifugation or microfiltration of the cheese milk, whereas the addition of nitrate, lysozyme, bacteriocins, or jenny milk may hamper germination and outgrowth of clostridia [23,24,25,26,27,28,29].

### 3.3. Organic Acid Concentrations and pH Values

**Figure 2:**
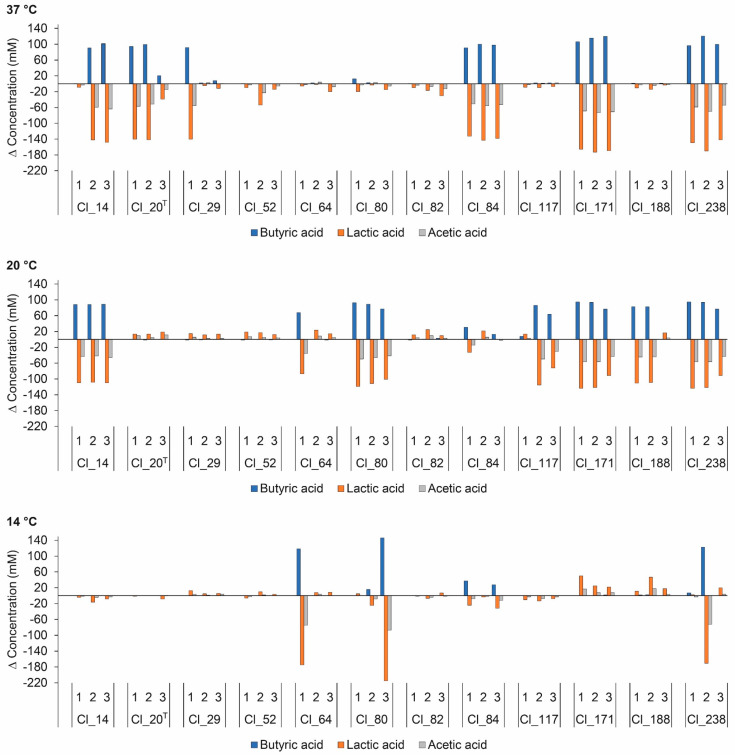
Changes in butyric, lactic, and acetic acid concentrations in enriched milk medium (EM) inoculated with *C. tyrobutyricum* strains after anaerobic incubation at 37, 20, and 14 °C for 5, 28, and 120 days, respectively. The number under each bar grouping indicates an individual replicate. ^T^ Type strain.

Organic acids are important characteristics associated with cheese manufacture and ripening, and, hence, with final cheese composition and quality [30]. Organic acid production may arise in cheese as a result of hydrolysis of milk fat during lipolysis, metabolism of residual lactose, citrate, and lactate, and bacterial growth [31]. Information on the changes in organic acid concentrations is important for the understanding of bacterial metabolism. Figure 2 depicts changes in organic acid concentrations in EM inoculated with different *C. tyrobutyricum* strains. The butyric acid concentration was negatively correlated with lactic acid concentration (*r* = −0.680). *C. tyrobutyricum* produces butyric acid from lactic acid via the Embden–Meyerhof–Parnas pathway. The fermentation pathway results in 1 mol of butyric acid, 2 mol of H_2_, and 2 mol of CO_2_ per 2 mol of lactic acid [32]. Hence, it is not surprising that the butyric acid concentration was positively correlated with gas production (*r* = 0.860). A similar observation was reported by Driehuis et al. for BB medium inoculated with *C. tyrobutyricum* strains [33]. The concentrations of organic acids, however, were lower compared to the concentrations obtained within this study. The difference in results could possibly be attributed to lower concentrations of sodium lactate (5.0 g/L) and sodium acetate (5.0 g/L) in BB medium.

The acetic acid concentration was positively correlated with lactic acid concentration (*r* = 0.949). The observed decrease in acetic acid concentration in replicates with positive gas production indicates its utilization by *C. tyrobutyricum*. Acetic acid is an intermediate on the pathway of lactic acid transformation to butyric acid and can be converted to acetyl-CoA, resulting in higher butyric acid yield [34]. In a study carried out in BB medium, Driehuis et al. reported conversion of acetic acid into butyric acid [33]. Cheeses contaminated with *C. tyrobutyricum* showed lower levels of acetic acid than control cheese, as reported by Gómez-Torres et al. [35]. On the contrary, Garde et al. reported production of acetic acid in milk inoculated with *C. tyrobutyricum* strains [12]. Furthermore, raw and pasteurized milk Manchego cheeses with LBD showed higher levels of acetic acids than cheeses without LBD [9], suggesting their production by *C. tyrobutyricum*.

Propionic acid was detected in very low concentrations within the range of 0.493–5.81 mM (detailed data not shown). However, concentrations of propionic acids were weakly positively correlated with gas production (*p* < 0.01; *r* = 0.574). Similar results were obtained by Garde et al. for milk contaminated with *C. tyrobutyricum* isolates under Manchego cheese ripening conditions [12].

Incubation at 14 °C resulted in significantly (*p* < 0.05) lower changes in organic acid concentrations compared to 37 and 20 °C. However, no significant difference in changes in organic acid concentrations was observed between 37 and 20 °C. These results are consistent with the previous observation that gas production was delayed at temperatures below 20 °C. This observation emphasizes that ripening temperatures have a critical effect on organic acid production and cheese quality.

**Figure 3:**
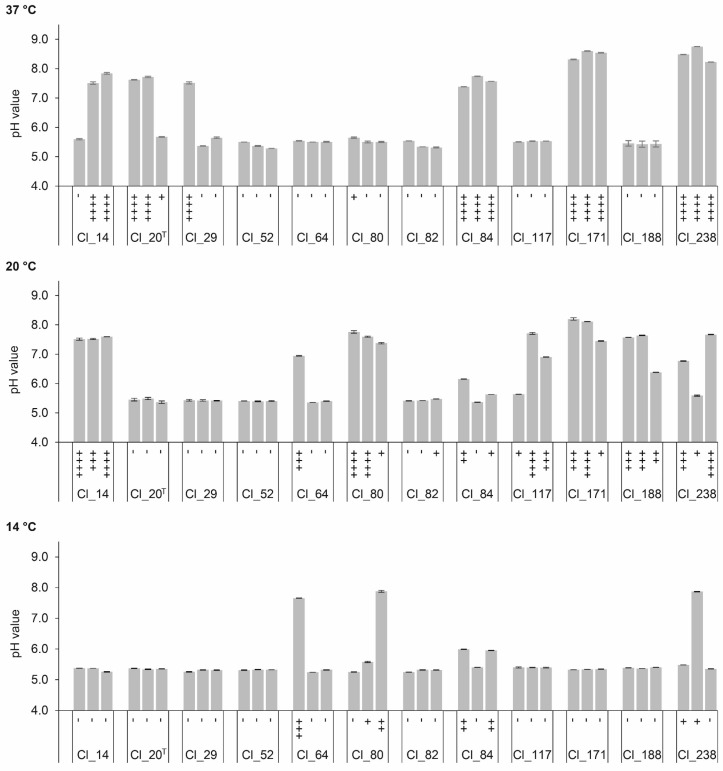
pH values and gas production in enriched milk medium (EM) inoculated with *C. tyrobutyricum* strains after anaerobic incubation at 37, 20, and 14 °C for 5, 28, and 120 days, respectively. The graph shows means of pH values ± standard deviations (*n* = 2). Gas production of each replicate is indicated under each bar and expressed as the height of plug displacement in cm: (-) <0.5 cm; (+) 0.5–2.9 cm; (++) 3.0–5.9 cm; (+++) 6.0–8.9 cm; (++++) 9.0–10.0 cm. ^T^ Type strain.

The conversion of organic acid during ripening of cheese also influences the cheese pH. The extent of pH change is determined by the amount of organic acid produced and the buffering capacity of the cheese [36]. Figure 3 depicts the changes in pH values of EM inoculated with *C. tyrobutyricum* strains. Correlational analysis indicated a positive correlation between the increase of pH and the amount of gas produced at all temperatures (*p* < 0.01, *r* = 0.844). The pH change was, as expected, also positively correlated with butyric acid concentration (*r* = 0.813). A pH increase was reported in LBD cheeses made from milk artificially contaminated with clostridial spores [5,37,38], as well as cheeses with natural occurring LBD [9]. The pH increase is assigned to deacidification arising from the metabolic activity of *Clostridium* [5,9,27,35]. Whereas, for most of the strains, the extent of pH change was in good agreement with the amount of gas produced, pH values of replicates of strain Cl_80 at 20 °C were not significantly different (*p* < 0.01), although one out of the three replicates significantly differed in gas production.

### 3.4. Inoculum Concentration

**Figure 4:**
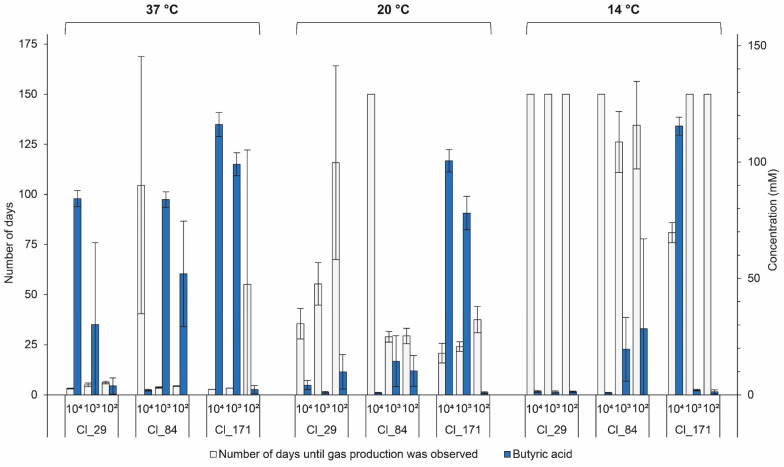
Number of days until gas production and butyric acid concentration in enriched milk medium (EM) inoculated with *C. tyrobutyricum* strains Cl_29, Cl_84, and Cl_171 at three different inoculation levels (10^4^, 10^3^, and 10^2^ spores/mL EM) during anaerobic incubation at 37, 20, and 14 °C. Butyric acid concentration was determined after 5, 28, and 120 days of anaerobic incubation at 37, 20, and 14 °C, respectively. Each bar represents the mean ± standard deviation (*n* = 3).

The influence of the inoculum concentration of strains Cl_29, Cl_84, and Cl_171 on the extent of butyric acid fermentation in EM is presented in Figure 4. A significant (*p* < 0.01) delay in gas production in two tested *C. tyrobutyricum* strains, namely, Cl_29 and Cl_171, with reduced inoculum concentration was observed. Accordingly, the butyric acid concentration was higher in EM with increased inoculum concentration. This is in good agreement with the observation of Silvetti et al. who reported faster gas production with higher inoculum levels of *C. tyrobutyricum* under similar conditions [10]. The effect of inoculum on the rate of germination and time to germination has been observed for *Clostridium botulinum* [39], *Bacillus stearothermophilus* [40], and *Bacillus megaterium* [41]. An explanation may be that spores interact with each other and, as they germinate, they release molecules that trigger germination of their dormant neighbors [42]. This was further supported by reports in which growth parameters derived from large inocula were used to stimulate growth from small numbers of spores. The stimulated times to detectable growth were smaller and more homogeneous than the observed detection times, suggesting the communication between the spores [40,43]. We also consider the possibility that the bacterial cells could be using a quorum-sensing mechanism to promote the germination, as reported for *C. botulinum* [44]. However, to the best of our knowledge, no studies have investigated quorum sensing in *C. tyrobutyricum*.

Surprisingly, the strain Cl_84 showed a different growth behavior when inoculated at the highest inoculum concentration. In fact, only one out of three replicates inoculated with 10^4^ spores/mL of EM showed gas production at 37 °C, and no gas production was visible at this concentration at 20 and 14 °C. However, at inoculum levels of 10^3^ and 10^2^ spores/mL of EM, the results coincided with the results of Cl_29 and Cl_171 insofar as delayed gas production with reduced inoculum concentration was observed at 37, 20, and 14 °C. When the experiment of strain Cl_84 was repeated in EM at 37 °C, previous results were confirmed as no gas production was observed for the undiluted spore suspension (10^4^ spores/mL of EM) but all tubes containing 10^3^ and 10^2^ spores/mL of EM showed gas production. Furthermore, the experiment at 37 °C was repeated in another medium, namely, reinforced clostridial medium (RCM, Merck, Germany), at inoculum levels of 10^3^, 10^4^, and 10^5^ spores/mL of RCM. Again, previous results were confirmed as no gas production was observed at an inoculum level of 10^4^ spores/mL of RCM. However, gas production at a higher concentration (10^5^ spores/mL of RCM), as well as at a lower level of 10^3^ spores/mL of RCM, was observed. According to current knowledge, we have no explanation for this germination or growth inhibition effect of strain Cl_84 at the concentration of 10^4^ spores/mL of medium. 

Overall, the results of this study indicate that the extent of butyric acid fermentation is not only dependent on inoculum concentrations but also driven by characteristics of the individual strains. This hypothesis is also supported by the fact that two of the four strains that produced gas at 14 °C, namely, Cl_64 and Cl_80 (7.2 × 10^1^ and 8.2 × 10^2^ spores/mL of EM, respectively), were inoculated in lower inoculum concentrations compared to other strains that did not produce gas at this temperature.

### 3.5. Phenotypic Diversity

The capability to produce gas and organic acids is considered to be a characteristic of *C. tyrobutyricum* [3,5,6,45]. Several differences in growth behavior, as well as gas and organic acid production, have been observed that cannot be exclusively attributed to different inoculum levels but rather to strain-dependent variability.

For instance, gas production of the type strain Cl_20 at 37 °C was faster, but growth at 20 °C was significantly slower compared to other strains, and, at 14 °C, no growth was detected at all. This observation suggests that the type strain, in contrast to milk and cheese isolates, may not be adapted to lower temperatures and may be displaying a less active metabolism than wild-type isolates. Another strain, namely, Cl_29, also showed faster outgrowth at 37 °C, but slower growth at 20 °C in comparison to other strains and no growth at all at 14 °C. This is in good agreement with the highly similar genotypes of strains Cl_20 and Cl_29 obtained using hexaplex-PCR typing (Figure 1b). Moreover, these two strains were grouped into the same cluster in the dendrogram according to MALDI-TOF MS spectra (Figure 1a). Furthermore, strain Cl_14 also showed fast outgrowth at 37 °C and no growth at 14 °C, but faster growth at 20 °C compared to Cl_20 and Cl_29. This strain also clustered in proximity to Cl_20 in both dendrograms in Figure 1a,b.

In contrast to Cl_20, Cl_29, and Cl_14, several milk and cheese isolates showed slower growth at 37 °C than other strains, but faster growth at 20 °C (Cl_117, Cl_188, Cl_64, and Cl_80). Indeed, Cl_64 and Cl_80 were among the four strains that were able to produce gas at 14 °C within the 170 day test period indicating extraordinary adaptation to low temperatures. Although Cl_64 and Cl_80 showed similar phenotypic characteristics, low similarity was observed between their MALDI-TOF MS spectra, as well as their hexaplex-PCR fingerprints (Figure 1a,b). Interestingly, Cl_64 clustered separately from other strains in the MALDI-TOF MS analysis, but showed high similarity with many other isolates in hexaplex-PCR fingerprinting (Figure 1b).

Only four out of 12 strains (Cl_64, Cl_80, Cl_84, and Cl_238) were able to produce gas at each of the three tested temperatures. However, genetic similarity did not become apparent within this group in the dendrogram.

Although two strains (Cl_82 and Cl_84) were isolated from raw milk obtained from the same production location, Cl_82 showed slower gas production at 20 °C compared to Cl_84 and no gas production at all at 37 and 14 °C. These two strains were also distributed in different clusters in the dendrogram according to hexaplex-PCR fingerprints but fell within the same cluster in the MALDI-TOF MS analysis (Figure 1a,b). Strain Cl_238 was isolated from the same production location as Cl_82 and Cl_84, but 1 year earlier. Within both dendrograms, it clustered separately from Cl_82 and Cl_84.

One strain, namely, Cl_52, did not show any metabolic activity at all. Even when the concentration of the spore inoculum was determined, this strain did not produce colonies on RCA (Table 1). Nevertheless, strain Cl_52 was included into the experiment due to its characteristic growth behavior in preliminary experiments (data not shown). Cl_52 had exhibited slower growth than other *C. tyrobutyricum* strains on RCA (5 days compared to 2 days). Furthermore, this strain clustered far from most of the other strains using the two typing methods within this study (Figure 1a,b) and GTG-5 typing [8]. To ensure the presence of the microorganism, phase-contrast microscopy of the spore suspension was performed and revealed that, indeed, the overall cell density of the spore suspension was lower compared to other strains of the test panel. Furthermore, the bacterium also appeared to be in a different state (Figure S1d, Supplementary Materials). Spores of Cl_52 were mostly visible within the vegetative cells suggesting that the development of free spores may not have been completed under the chosen incubation conditions. For another strain (Cl_82), no colonies were produced on RCA when the concentration of the spore suspension was determined. The presence of spores, however, was confirmed using phase-contrast microscopy, in which a higher number of vegetative cells than spores was observed (Figure S1g, Supplementary Materials).

The results of this study show that different strains of *C. tyrobutyricum* vary in their ability to form spores and subsequently germinate in vitro. Our findings are consistent with those on *C. botulinum,* i.e., that germination is dependent on the strain, presumably due to the strain-specific germinant specificity [46]. One possible explanation for the high heterogeneity among the tested strains could be impaired germination of spores rather than decreased ability of germinated spores to form butyric acid and gases [3].

The fact that spore formation is strictly related to environmental stress may affect the ability of *C. tyrobutyricum* to germinate and grow at lower temperatures [47]. While many studies clarified the conditions that affect *C. tyrobutyricum* growth at reduced temperatures, no work has been done to characterize the specific molecular components and their roles that allow such growth to occur [48,49,50]. It is likely that, according to the mechanisms of other related spore-formers, membrane fatty acid components are altered to increase fluidity, and RNA stability mechanisms are involved [51]. The obtained results indicate that some strains may be adapted to lower temperatures, suggesting strain-specific adaptation mechanisms.

However, although we observed several strain-dependent effects, we also noticed great variation in butyric acid fermentation ability within the replicates of the same strain despite a homogeneous and a common inoculum. For instance, only two of three replicates of strain Cl_64 produced gas at 37 °C, while only one of three replicates produced gas at 14 °C (Table 2). Furthermore, particularly replicates of strains that were inoculated at low concentrations (Cl_64, Cl_80, Cl_82, Cl_117) exhibited high heterogeneity in time spans until gas production was observed. Possible reasons for the observed heterogeneity may be distinct amounts of germination receptors per spore, germination commitment, or the subsequent stages of germination, outgrowth, and vegetative growth [49,52,53]. Because a greater percentage of rapid-germinating spores is expected at higher spore densities, the apparent spore germination of a population may be associated with inoculum size [41]. It is also possible that a small fragment of spore population, termed superdormant spores, may exhibit extremely long and unpredictable germination and outgrowth responses or not respond to known germination triggers [49]. Previous studies on different *Clostridium* species, *C. perfringens*, *C. botulinum*, and *Clostridioides* (*Clostridium) difficile,* demonstrated that the presence of superdormant spores also contributes to germination heterogeneity [50,54,55,56]. To the best of our knowledge, no work has been focused on *C. tyrobutyricum*.

On the basis of the varying germination behaviors observed in this study, we conclude that some of the tested strains may pose a higher risk for cheese spoilage than others. Four strains (Cl_64, Cl_80, Cl_84, and Cl_238) germinated and produced gas at each of the tested temperatures including the temperature of 14 °C. As this temperature is close to the temperatures occurring during cheese ripening, this group of strains may constitute a high risk for late blowing defects. However, it is important to point out that strain Cl_84 did not produce gas at the spore concentration of 10^4^ spores/mL of EM but at every other tested concentration level. Some strains (Cl_14, Cl_117, Cl_171, and Cl_188) may be considered as an “intermediate risk” because these strains did not grow at 14 °C but showed good growth at 20 °C. A low risk for cheese spoilage is expected from strains Cl_20 and Cl_29, as well as Cl_82, because these strains showed no growth at 14 °C and slow outgrowth at 20 °C. Because Cl_52 showed no metabolic activity in any of the tested conditions, this strain seems to pose a very low risk for cheese spoilage. Of course, it is important to consider that the spoilage potential of the tested strains may be different in cheese for several reasons. Raw milk and cheese, for instance, provide a complex habitat for different microorganisms that may interact with clostridia and vice versa. Several authors highlighted the synergistic effect between clostridial species and/or strains [3,5,32], whereas lactobacilli and some related bacteria may inhibit the growth of *C. tyrobutyricum* by producing different antibiotic compounds [26,57].

## 4. Conclusions

In conclusion, the present study supports the hypothesis that cheese spoilage may be not only dependent on clostridial spore concentrations but also on characteristics of individual *C. tyrobutyricum* strains. Across the tested strain set, we observed high genotypic and moderate proteotypic diversity. Some of the genotypic and proteotypic differences and similarities also manifested themselves in phenotypic traits, such as germination and gas formation at different temperatures. However, variations were not only detected among the tested strains but also among replicates of the same strain. This indicates that differences within populations of the same strain and developmental stages of single cells and spores also may be relevant.

This study provides interesting insights into strain-dependent germination and gas formation of *C. tyrobutyricum*. The results indicate that some strains of this species may be more relevant for cheese spoilage than others. The findings obtained in this experimental setting may serve as a basis for future studies in cheese. In addition, genomic and transcriptomic research is encouraged to enhance our understanding of the genetic background of the observed phenotypic differences among *C. tyrobutyricum* strains.

## Figures and Tables

**Table 1:** *Clostridium tyrobutyricum* strains used in this study.

Strain Number	Strain Collection ^a^ and Number	Origin	Production Location ^b^	Year of Isolation	Spore Concentration in Original Spore Suspension (CFU/mL)	Spores per mL Enriched Milk Medium
Cl_14	DSM 663	Emmental cheese	n.a.	before 1964	5.1 × 10^2^	1.0 × 10^2^
Cl_20 ^T^	DSM 2637	n.a.	n.a.	before 1971	3.2 × 10^3^	6.4 × 10^2^
Cl_29	NIZO BZ 15	n.a.	n.a.	1970	2.1 × 10^5^	4.2 × 10^3^
Cl_52	FAM25158	Emmental cheese	n.a.	1966	<10	<10
Cl_64	BOKU M33	Raw milk	C	2016	3.6 × 10^2^	7.2 × 10^1^
Cl_80	BOKU M9	Raw milk	A	2016	4.1 × 10^3^	8.2 × 10^2^
Cl_82	BOKU M12	Raw milk	B	2016	<10	<10
Cl_84	BOKU M17	Raw milk	B	2016	7.0 × 10^4^	1.4 × 10^3^
Cl_117	BOKU M59	Raw milk	A	2016	3.1 × 10^2^	6.2 × 10^1^
Cl_171	BOKU K13	Semihard cheese	G	2015	9.5 × 10^4^	1.9 × 10^3^
Cl_188	BOKU K47	Cheese–sausage mixture	E	2015	7.6 × 10^3^	1.5 × 10^3^
Cl_238	BOKU K28	Hard cheese	B	2015	2.6 × 10^4^	5.2 × 10^3^

^a^ DSM, Deutsche Sammlung von Mikroorganismen und Zellkulturen GmbH (German Collection of Microorganisms and Cell Cultures), Braunschweig, Germany; NIZO, NIZO food research, Ede, The Netherlands; BOKU, University of Natural Resources and Life Sciences Vienna, Austria. ^b^ Letters representing different production locations. ^T^ Type strain. CFU, colony-forming units; n.a., data not available.

**Table 2:** Numbers of days of incubation required to observe a plug displacement greater than 0.5 cm caused by gas production of *C. tyrobutyricum* strains in enriched milk medium (EM) during anaerobic incubation at 37 °C for 150 days, and at 20 and 14 °C for 170 days.

Strain	Temperature		
	37 °C	20 °C	14 °C
Cl_14	5.5	23.7	-
	3.3	22.8	-
	4	22.8	-
Cl_20 ^T^	3.3	39.7	-
	3.4	34.8	-
	4.7	38.6	-
Cl_29	3.8	48.7	-
	5.9	70.3	-
	5.4	47	-
Cl_52	-	-	-
	-	-	-
	-	-	-
Cl_64	5.8	25.9	107.2
	12.9	40	-
	-	34.8	-
Cl_80	4.8	21.9	-
	6.3	22.9	115.8
	7.4	26.8	73.8
Cl_82	-	39	-
	-	43.8	-
	-	27.7	-
Cl_84	3.5	27.4	115.3
	3.5	32.6	147.7
	4.2	26.8	115.3
Cl_117	7.6	27.7	-
	8.5	23.8	-
	10.5	26	-
Cl_171	3.4	22.1	-
	3.5	22.6	-
	3.4	27.4	-
Cl_188	7.5	22.6	-
	7.9	23.9	-
	8.3	26.4	-
Cl_238	3.7	25.9	109.7
	3.3	26.8	80.6
	3.9	24.3	-

-, No gas production observed by the end of the incubation period. ^T^ Type strain.

## Supplementary Materials

The following are available online at https://www.mdpi.com/2076-2607/8/11/1836/s1: Figure S1. Phase-contrast microscope images of 12 spore suspensions of *C. tyrobutyricum* strains: (a) Cl_14; (b) Cl_20 (type strain; dilution 1:10); (c) Cl_29; (d) Cl_52; (e) Cl_64 (dilution 1:10); (f) Cl_80 (dilution 1:10); (g) Cl_82 (dilution 1:10); (h) Cl_84; (i) Cl_117; (j) Cl_171 (dilution 1:10); (k) Cl_188 (dilution 1:10); and (l) Cl_238. The spore suspensions are observed under 1000× magnification with oil under a phase-contrast microscope. Scale bars indicate 10 µm.

## References

[1] Brändle J., Domig K.J., Kneifel W. (2016). Relevance and analysis of butyric acid producing clostridia in milk and cheese. Food Control.

[2] Doyle C.J., Gleeson D., Jordan K., Beresford T.P., Ross R.P., Fitzgerald G.F., Cotter P.D. (2015). Anaerobic sporeformers and their significance with respect to milk and dairy products. Int. J. Food Microbiol..

[3] Klijn N., Nieuwenhof F.F.J., Hollwerf J.D., Vanderwaals C.B., Weerkamp A.H. (1995). Identification of *Clostridium tyrobutyricum* as the causative agent of late blowing in cheese by species-specific PCR amplification. Appl. Environ. Microbiol..

[4] Julien M.C., Dion P., Lafreniere C., Antoun H., Drouin P. (2008). Sources of clostridia in raw milk on farms. Appl. Environ. Microbiol..

[5] Le Bourhis A.-G., Doré J., Carlier J.-P., Chamba J.-F., Popoff M.-R., Tholozan J.-L. (2007). Contribution of *C. beijerinckii* and *C. sporogenes* in association with *C. tyrobutyricum* to the butyric fermentation in Emmental type cheese. Int. J. Food Microbiol..

[6] Brändle J., Fraberger V., Berta J., Puglisi E., Jami M., Kneifel W., Domig K.J. (2018). Butyric acid producing clostridia in cheese—Towards the completion of knowledge by means of an amalgamate of methodologies. Int. Dairy J..

[7] Bermúdez J., González M.J., Olivera J.A., Burgueño J.A., Juliano P., Fox E.M., Reginensi S.M. (2016). Seasonal occurrence and molecular diversity of clostridia species spores along cheesemaking streams of 5 commercial dairy plants. J. Dairy Sci..

[8] Burtscher J., Küller F., Dreier M., Arias-Roth E., Drissner D., Domig K.J. (2020). Characterization of *Clostridium tyrobutyricum* strains using three different typing techniques. Microorganisms.

[9] Garde S., Ávila M., Gaya P., Arias R., Nuñez M. (2012). Sugars and organic acids in raw and pasteurized milk Manchego cheeses with different degrees of late blowing defect. Int. Dairy J..

[10] Silvetti T., Morandi S., Brasca M. (2018). Growth factors affecting gas production and reduction potential of vegetative cell and spore inocula of dairy-related *Clostridium* species. LWT.

[11] Ruusunen M., Surakka A., Korkeala H., Lindström M. (2012). *Clostridium tyrobutyricum* strains show wide variation in growth at different NaCl, pH, and temperature conditions. J. Food Prot..

[12] Garde S., Arias R., Gaya P., Nuñez M. (2011). Occurrence of *Clostridium* spp. in ovine milk and Manchego cheese with late blowing defect: Identification and characterization of isolates. Int. Dairy J..

[13] Brändle J., Heinzle L., Fraberger V., Berta J., Zitz U., Schinkinger M., Stocker W., Kneifel W., Domig K.J. (2018). Novel approach to enumerate clostridial endospores in milk. Food Control.

[14] D’Incecco P., Pellegrino L., Hogenboom J.A., Cocconcelli P.S., Bassi D. (2018). The late blowing defect of hard cheeses: Behaviour of cells and spores of *Clostridium tyrobutyricum* throughout the cheese manufacturing and ripening. LWT.

[15] Vorarlberger Bergkäse g.U. Annex I Council Regulation (EEC) No 2081/92. https://ec.europa.eu/agriculture/quality/door/registeredName.html?denominationId=777.

[16] Tiroler Bergkäse g.U. Council Regulation (EEC) No 2081/92. https://ec.europa.eu/agriculture/quality/door/registeredName.html?denominationId=705.

[17] Guinee T.P., Fox P.F., McSweeney P.L.H., Fox P.F., Cotter P.D., Everett D.W. (2017). Chapter 13—Salt in Cheese: Physical, Chemical and Biological Aspects. Cheese.

[18] Rainey F.A., Hollen B.J., Small A.M., Whitman W.B., Rainey F., Kämpfer P., Trujillo M., Chun J., DeVos P., Hedlund B., Dedysh S. (2015). Clostridium. Bergey’s Manual of Systematics of Archaea and Bacteria.

[19] Bachmann H.P., Bütikofer U., Fröhlich-Wyder M.T., Isolini D., Jakob E., Fuquay J.W. (2011). Cheese | Swiss-Type Cheeses. Encyclopedia of Dairy Sciences.

[20] Su Y.-C., Ingham S.C. (2000). Influence of milk centrifugation, brining and ripening conditions in preventing gas formation by *Clostridium* spp. in Gouda cheese. Int. J. Food Microbiol..

[21] Morandi S., Battelli G., Silvetti T., Tringali S., Nunziata L., Villa A., Acquistapace A., Brasca M. (2021). Impact of salting and ripening temperatures on late blowing defect in Valtellina Casera PDO cheese. Food Control.

[22] Burtscher J., Hobl L., Kneifel W., Domig K.J. (2020). Short communication: Clostridial spore counts in vat milk of Alpine dairies. J. Dairy Sci..

[23] Arias C., Oliete B., Seseña S., Jimenez L., Pérez-Guzmán M.D., Arias R. (2013). Importance of on-farm management practices on lactate-fermenting *Clostridium* spp. spore contamination of Manchega ewe milk: Determination of risk factors and characterization of *Clostridium* population. Small Rumin. Res..

[24] Zucali M., Bava L., Colombini S., Brasca M., Decimo M., Morandi S., Tamburini A., Crovetto G.M. (2015). Management practices and forage quality affecting the contamination of milk with anaerobic spore-forming bacteria. J. Sci. Food Agric..

[25] Klantschitsch T. (1999). Influence of Microfiltration on the Quality of Semi-Hard Cheese from Raw Milk with Particular Emphasis on *Clostridium tyrobutyricum* Spores. Ph.D.Thesis.

[26] Ávila M., Gómez-Torres N., Hernández M., Garde S. (2014). Inhibitory activity of reuterin, nisin, lysozyme and nitrite against vegetative cells and spores of dairy-related *Clostridium* species. Int. J. Food Microbiol..

[27] Bogovič Matijašić B., Koman Rajšp M., Perko B., Rogelj I. (2007). Inhibition of *Clostridium tyrobutyricum* in cheese by *Lactobacillus gasseri*. Int. Dairy J..

[28] Cosentino C., Paolino R., Valentini V., Musto M., Ricciardi A., Adduci F., D’Adamo C., Pecora G., Freschi P. (2015). Effect of jenny milk addition on the inhibition of late blowing in semihard cheese. J. Dairy Sci..

[29] Stadhouders J. (1990). Prevention of butyric acid fermentation by the use of nitrate. Bull. Int. Dairy Fed..

[30] Fox P.F., Lucey J.A., Cogan T.M. (1990). Glycolysis and related reactions during cheese manufacture and ripening. Crit. Rev. Food Sci. Nutr..

[31] Fox P.F., Law J., McSweeney P.L.H., Wallace J., Fox P.F. (1993). Biochemistry of Cheese Ripening. Cheese: Chemistry, Physics and Microbiology: Volume 1 General Aspects.

[32] Jakob E. (2005). Buttersäureblähung-noch immer aktuell. Proc. ALP Forum.

[33] Driehuis F., Hoolwerf J., Rademaker J.L.W. (2016). Concurrence of spores of *Clostridium tyrobutyricum, Clostridium beijerinckii* and *Paenibacillus polymyxa* in silage, dairy cow faeces and raw milk. Int. Dairy J..

[34] Lee J., Jang Y.-S., Han M.-J., Kim J.Y., Lee S.Y. (2016). Deciphering *Clostridium tyrobutyricum* metabolism based on the whole-genome sequence and proteome analyses. mBio.

[35] Gómez-Torres N., Garde S., Peirotén A., Ávila M. (2015). Impact of *Clostridium* spp. on cheese characteristics: Microbiology, color, formation of volatile compounds and off-flavors. Food Control.

[36] Lucey J.A., Fox P.F. (1993). Importance of calcium and phosphate in cheese manufacture: A review. J. Dairy Sci..

[37] Borreani G., Ferrero F., Nucera D., Casale M., Piano S., Tabacco E. (2019). Dairy farm management practices and the risk of contamination of tank milk from *Clostridium* spp. and *Paenibacillus* spp. spores in silage, total mixed ration, dairy cow feces, and raw milk. J. Dairy Sci..

[38] Garde S., Ávila M., Arias R., Gaya P., Nuñez M. (2011). Outgrowth inhibition of *Clostridium beijerinckii* spores by a bacteriocin-producing lactic culture in ovine milk cheese. Int. J. Food Microbiol..

[39] Zhao L., Montville T.J., Schaffner D.W. (2000). Inoculum size of *Clostridium botulinum* 56A spores influences time-to-detection and percent growth-positive samples. J. Food Sci..

[40] Llaudes M.K., Zhao L., Duffy S., Schaffner D.W. (2001). Simulation and modelling of the effect of small inoculum size on time to spoilage by *Bacillus stearothermophilus*. Food Microbiol..

[41] Caipo M.L., Duffy S., Zhao L., Schaffner D.W. (2002). *Bacillus megaterium* spore germination is influenced by inoculum size. J. Appl. Microbiol..

[42] Webb M.D., Stringer S.C., Le Marc Y., Baranyi J., Peck M.W. (2012). Does proximity to neighbours affect germination of spores of non-proteolytic *Clostridium botulinum*?. Food Microbiol..

[43] Zhao L., Montville T.J., Schaffner D.W. (2003). Computer simulation of *Clostridium botulinum* strain 56A behavior at low spore concentrations. Appl. Environ. Microbiol..

[44] Zhao L., Montville T.J., Schaffner D.W. (2006). Evidence for quorum sensing in *Clostridium botulinum* 56A. Lett. Appl. Microbiol..

[45] Le Bourhis A.-G., Saunier K., Doré J., Carlier J.-P., Chamba J.-F., Popoff M.-R., Tholozan J.-L. (2005). Development and validation of PCR primers to assess the diversity of *Clostridium* spp. in cheese by temporal temperature gradient gel electrophoresis. Appl. Environ. Microbiol..

[46] Alberto F., Broussolle V., Mason D.R., Carlin F., Peck M.W. (2003). Variability in spore germination response by strains of proteolytic *Clostridium botulinum* types A, B and F. Lett. Appl. Microbiol..

[47] Tanner F.W., Oglesby E.W. (1936). Influence of temperature on growth and toxin production by *Clostridium botulinum*. J. Food Sci..

[48] Setlow P., Liu J., Faeder J.R., Abel-Santos A. (2012). Heterogeneity in bacterial spore populations. Bacterial Spores: Current Research and Applications.

[49] Setlow P., Wang S., Li Y.-Q. (2017). Germination of spores of the orders Bacillales and Clostridiales. Annu. Rev. Microbiol..

[50] Stringer S.C., Webb M.D., Peck M.W. (2011). Lag time variability in individual spores of *Clostridium botulinum*. Food Microbiol..

[51] Checinska A., Paszczynski A., Burbank M. (2015). *Bacillus* and other spore-forming genera: Variations in responses and mechanisms for survival. Annu. Rev. Food Sci. Technol..

[52] Luu S., Setlow P. (2014). Analysis of the loss in heat and acid resistance during germination of spores of *Bacillus* species. J. Bacteriol..

[53] Pandey R., Ter Beek A., Vischer N.O.E., Smelt J.P.P.M., Brul S., Manders E.M.M. (2013). Live cell imaging of germination and outgrowth of individual *Bacillus subtilis* spores; the effect of heat stress quantitatively analyzed with SporeTracker. PLoS ONE.

[54] Deng K., Talukdar P.K., Sarker M.R., Paredes-Sabja D., Torres J.A. (2017). Survival of *Clostridium difficile* spores at low water activity. Food Microbiol..

[55] Wang G., Paredes-Sabja D., Sarker M.R., Green C., Setlow P., Li Y.-Q. (2012). Effects of wet heat treatment on the germination of individual spores of *Clostridium perfringens*. J. Appl. Microbiol..

[56] Zhang Y., Mathys A. (2019). Superdormant spores as a hurdle for gentle germination-inactivation based spore control strategies. Front. Microbiol..

[57] Rilla N., Martinez B., Delgado T., Rodriguez A. (2003). Inhibition of *Clostridium tyrobutyricum* in Vidiago cheese by *Lactococcus lactis* ssp. *lactis* IPLA 729, a nisin Z producer. Int. J. Food Microbiol..

