# Supplementary material for: Strain-Dependent Cheese Spoilage Potential of Clostridium tyrobutyricum
Source: Microorganisms. 2020 Nov 22;8(11):1836. doi: 10.3390/microorganisms8111836 (PMC7700369; doi:10.3390/microorganisms8111836)
Supplement: Supplementary file 1 [file microorganisms-08-01836-s001.zip › Supplementary Materials.pdf]

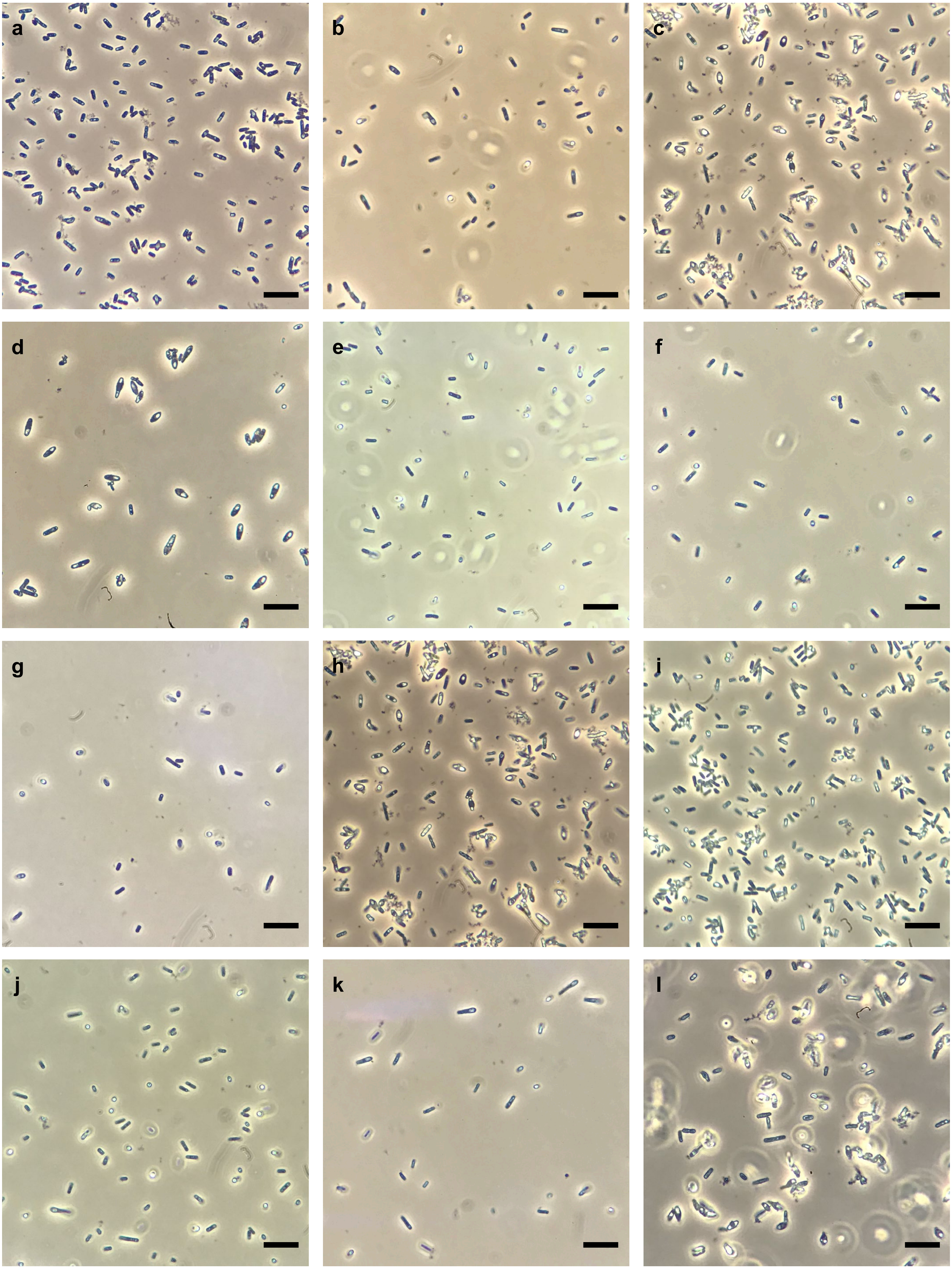

Figure S1. Phase-contrast microscope images of 12 spore suspensions of *C. tyrobutyricum* strains as follows: (a) Cl_14; (b) Cl_20 (type strain; dilution 1:10); (c) Cl_29; (d) Cl_52; (e) Cl_64 (dilution 1:10); (f) Cl_80 (dilution 1:10); (g) Cl_82 (dilution 1:10); (h) Cl_84; (i) Cl_117; (j) Cl_171 (dilution 1:10); (k) Cl_188 (dilution 1:10); and (l) Cl_238. The spore suspensions are observed under 1000× magnification with oil under phase-contrast microscope. Scale bars indicate 10 μm.
